# Indocyanine green fluorescence applied to gynecologic oncology: beyond sentinel lymph node

**DOI:** 10.1097/JS9.0000000000001318

**Published:** 2024-03-15

**Authors:** Matteo Loverro, Nicolò Bizzarri, Filippo M. Capomacchia, Rafał Watrowski, Denis Querleu, Alessandro Gioè, Angelica Naldini, Francesco Santullo, Nazario Foschi, Anna Fagotti, Giovanni Scambia, Francesco Fanfani

**Affiliations:** a UOC Ginecologia Oncologica, Dipartimento di Scienze della Salute della Donna, del Bambino e di Sanità Pubblica, Fondazione Policlinico Universitario A. Gemelli, IRCCS; b Università Cattolica del Sacro Cuore; c Operational Unit of Peritoneum and Retroperitoneum Surgery, Fondazione Policlinico Universitario Agostino Gemelli IRCCS, Largo A. Gemelli; d Urology Division, Fondazione Policlinico Universitario “Agostino Gemelli” IRCCS, Rome, Italy; e Department of Gynecology and Obsterics, Helios Hospital Müllheim, Teaching Hospital of the University of Freiburg, 79379 Müllheim; f Faculty of Medicine, Medical Center - University of Freiburg, 79106 Freiburg, Germany

**Keywords:** angiography, cervical cancer, endometrial cancer, gynecologic oncology, indocyanine green, lymphography, ovarian cancer, theranostic, vulvar cancer

## Abstract

Indocyanine green (ICG), a well-known molecule employed in medicine for over five decades, has emerged as a versatile dye widely embraced across various surgical disciplines. In gynecologic oncology, its prevalent use revolves around the detection of sentinel lymph nodes. However, the true potential of ICG extends beyond this singular application, owing to its pragmatic utility, cost-effectiveness, and safety profile. Furthermore, ICG has been introduced in the theranostic landscape, marking a significant juncture in the evolution of its clinical utility. This narrative review aims to describe the expanding horizons of ICG fluorescence in gynecologic oncology, beyond the sentinel lymph node biopsy. The manifold applications reported within this manuscript include: 1) lymphography; 2) angiography; 3) nerve visualization; 4) ICG-driven resections; and 5) theranostic. The extensive exploration across these numerous applications, some of which are still in the preclinical phase, serves as a hypothesis generator, aiming to stimulate the development of clinical studies capable of expanding the use of this drug in our field, enhancing the care of gynecological cancer patients.

## Introduction

Highlights

- Indocyanine green (ICG), a widely applied molecule for over five decades, has gained widespread acceptance in various surgical disciplines, particularly in gynecologic oncology.
- Its prevalent application in gynecologic oncology is the detection of sentinel lymph nodes.
- ICG’s potential transcends sentinel node detection due to its pragmatic use, cost-effectiveness, and favorable safety profile.
- We explored manifold applications of ICG fluorescence in gynecologic oncology, including lymphography, angiography, nerve visualization, ICG-driven resections, highlighting potential for broader adoption.
- ICG’s integration into the theranostic landscape represents a notable advancement, signifying its evolving clinical utility.

To ensure optimal oncological outcomes, surgeons must navigate the delicate balance of achieving macroscopic disease clearance while preserving critical functional structures. The advent of real-time fluorescence imaging has offered an invaluable insight into both anatomy and function, in surgical practice. Among the array of fluorescent tracers, indocyanine green (ICG) emerges as a reliable tool, facilitating intraoperative visualization through near-infrared cameras (NIR)^1,2^. Administered either intravenously or locally, ICG swiftly binds to lipoproteins, coursing through the bloodstream and lymphatic channels, with a brief half-life of 2–4 minutes, and undergoes hepatic metabolism^3^. Notably, its utilization is contraindicated in cases of pregnancy, iodine allergy, prior allergic reactions, or liver and kidney failure. Renowned for its safety profile and ease of application, ICG has become the most widely employed dye in both gynecological and general surgery.

In gynecologic oncology, ICG has predominantly found its niche in sentinel lymph node biopsy (SLNB), a subject that has already collected extensive reviews and in-depth investigations^4^. However, beyond the SLNB, ICG is a versatile tool, offering augmented reality insights into both anatomical structures^5^ and functional aspects^6^. Furthermore, recent developments have unveiled the therapeutic potential of ICG in photodynamic therapy, shedding new light on its multifaceted utility^7^.

The rationale of this work is to strive for the promotion of clinical studies utilizing ICG fluorescence, aiming to enhance the surgical and oncological outcomes for patients affected by ovarian carcinoma. Therefore, this review aim to unravel the breadth of ICG applications in gynecologic oncology, transcending the well-known use for SLNB. While many of these applications currently occupy a niche, with limited cases to their credit, they harbor untapped potential for broader integration in the surgery of tomorrow.

To facilitate readers’ navigation, the review has been divided based on the types of applications researchers have employed fluorescence for: 1) Lymphography, primarily used in preventing oncologic lymphedema and post-lymphadenectomy complications; 2) Angiography, employed to assess intraoperative vascularization in reconstructions of the bowel, ureters, vagina, urinary diversions, plastic flaps, and uterus transpositions/transplants, as well as for recognizing structures such as nerves and ureters; 3) Local and systemic administrations of the drug to intraoperatively guide the resection of anatomical structures and identify neoplastic tissues compared to healthy tissues; 4) Theranostics, namely the pioneering implementation of ICG in nanoprobes capable of extremely selective drug transport to tumor tissues.

## Materials and methods

Literature Search: To explore the diverse applications of ICG in gynecologic oncology, excluding SLNB, a comprehensive literature search was conducted up to March 2023. This search involved a double-blind approach conducted by the three authors: ML, NB, and RW.

Search Databases: We systematically searched several electronic databases, including PubMed, Web of Science, Google Scholar, and Scopus, using a set of predefined keywords. The keywords used for the search included ‘indocyanine green’, ‘ICG’, ‘near infra-red’, ‘fluorescence’, in combination with terms such as ‘gynecologic oncology’, ‘lymphography’, ‘angiography’, ‘anastomosis’, ‘anastomotic leakage’, ‘ureter’, ‘uterus’, and ‘vaginal cuff’.

Study Selection: All relevant articles were retrieved, and the respective reference lists were scrutinized to identify additional studies for potential inclusion. The shared criteria for deeming a publication relevant included the use of ICG in surgeries for gynecological tumors, the number of citations in other journals, and, in the case of publications from the same working group, a preference was given to more recent publications. Non-English language literature, duplicates, and abstracts without full text were excluded. Our review was guided by three strategic decisions. First, we exclusively included studies that explored the use of ICG in gynecological tumors, excluding those focused solely on nongynecological tumors. Second, considering the limited studies on the topic, we also selected case reports and case series with a restricted number of patients. Third, in instances where evidence in gynecologic oncology was limited but robust evidence existed, such as randomized clinical trials in other disciplines, these articles were subsequently included in the review. The identified publications were evenly distributed and underwent thorough examination by the authors. In cases of disagreements in the selection process, a final decision was reached through discussions involving two authors (M.L. and N.B.).

Results: The electronic database search yielded a total of 683 studies. After applying the inclusion and exclusion criteria, 564 papers were excluded as they did not meet the study’s objectives. Ultimately, 82 studies were deemed eligible for inclusion in this review.

## Lymphography

After the local injection of ICG, the molecule drains through the soft tissues and enters the lymphatic circulation, following its flow and allowing the fluorescent visualization of the lymphatic vessels (LVs). This process describes the anatomical course and provides information on the functional state of the lymphatic circulation. Plastic surgeons have extensively utilized this application for the treatment of lower limb lymphedema (LLLE), a common occurrence after surgery for gynecological tumors. Moreover, ICG lymphography has been employed intraoperatively to identify lesions of the LVs causing chylous ascites. In this section, studies related to the use of ICG for lymphography in patients with gynecological tumors will be analyzed.

LLLE is a complex clinical issue and one of the most challenging complications following inguinal and pelvic lymphadenectomy. Lymphedema is not solely related to fluid retention in the lower limb; it involves an inflammatory response, scarring of LVs, and reactive fat hypertrophy. These factors contribute to a spectrum of clinical presentations, ranging from fluid-dominant to fat hypertrophy/fibrosis-dominant lymphedema. Surgical treatment becomes necessary when conservative therapies are no longer effective^8^.

Various surgical approaches are available to address LLLE. Typically, fat/fibrosis-dominant limbs are treated with liposuction, while those with fluid-dominant, early-stage lymphedema may be candidates for lympho-venular anastomosis or lymphatic flap procedures. These techniques require preoperative or intraoperative lymphography to identify suitable lymphatic tissue donor sites or incision sites for anastomosis^8^.

Introduced by Unno *et al*. in 2007^9^, ICG lymphography is a non-invasive method that enables real-time assessment of lymphatic drainage. Different lymphatic patterns have been described during ICG lymphography, ranging from normal linear patterns to abnormal dermal backflow patterns. As the disease progresses, these patterns evolve from a splash pattern to stardust and diffuse patterns^10–13^. ICG lymphography allows LLLE to be classified into four stages, aiding decision-making^10,14,15^. The MD Anderson ICG scale, originally developed for upper limb lymphedema, has been adapted for LLLE^14,15^. Patients in stages III and IV, refractory to conservative treatments, may require surgical intervention^10^.

Suami *et al*.^16^ proposed a diagnostic protocol for identifying structural changes and guiding interventional strategies in cancer-related and noncancer-related LLLE. Through the analysis of 326 ICG lymphographies, they identified eight drainage regions. Among these, only two—the ipsilateral inguinal and popliteal regions—were original regions, while the remaining six were compensatory regions. The most common compensatory pathways include the contralateral inguinal region, identified in 13–30% of patients with cancer-related LLLE, followed by the gluteal pathway in 4–23%^16,17^.

Microsurgical lymphaticovenous anastomosis (LVA) is a bypass procedure that achieves lymphedema reduction by channeling stagnant lymph from the LVs via recipient veins in the lymphedematous limbs.

Caretto *et al*.^18^ performed LVA in 33 patients who had previously undergone pelvic lymphadenectomy for gynecological malignancies. Preoperative ICG was used to assess leg lymphatic drainage and plan incision sites for performing the anastomosis, resulting in a significant reduction in limb circumference after surgery.

ICG lymphography has also been employed to diagnose subclinical secondary lymphedema, aiding in the selection of patients eligible for early LVA^19^. Moreover, preoperative identification of subcutaneous LVs assists in the successful performance of LVA in LLLE patients^20,21^.

In another study, Yang *et al*.^22^ employed ICG lymphography, along with three other microscopic parameters, to assess the functionality of (LVA) conducted in a comprehensive retrospective series comprising 87 patients affected by gynecological cancer-related lymphedema. They employed a propensity score matching method to compare patients undergoing antegrade LVA with those undergoing both antegrade and retrograde anastomosis. The primary objective of the study, the percentage reduction of lymphedema at 6 and 12 months, appeared to be better, though not statistically significant, in the antegrade anastomosis-only group. Consequently, the findings of this study discourage the use of additional retrograde LVA.

Inguinal lymphadenectomy carries the highest risk of LLLE, leading some authors to suggest a preventive approach involving microsurgical reconstruction of lymphatic drainage following lymphadenectomy. Gentileschi *et al*.^23^ described a lymphatic flap to prevent LLLE after groin dissection, which, when guided by ICG lymphography, resulted in a significant difference between preoperative and postoperative limb volumes in treated versus untreated limbs.

In all these retrospective studies, the quality of evidence is limited by the small number of enrolled patients. However, the authors suggest that ICG lymphography is extremely valuable in diagnosing lymphedema, selecting patients, and defining surgical sites for performing LVAs.

Beyond inguinal lymphadenectomy, systematic pelvic and para-aortic lymphadenectomy is a staging procedure necessary in early-stage ovarian and cervical cancers^24^. Such procedures carry a significant risk of postoperative lymphatic complications^25^.

Iatrogenic injury to lymphatic pathways can lead to chylous ascites, observed in up to 33% of patients after para-aortic lymphadenectomy and occasionally after pelvic node dissection^26^. Although there is currently no evidence supporting the preventive role of indocyanine in iatrogenic lymphatic damage, ICG lymphography can play a role in recognizing and addressing chylous ascites that are not manageable with conservative therapy. Locating the fistula can be challenging due to postoperative inflammation and the size of LVs. Papadia *et al*.^27^ reported a case of ICG-guided surgical repair of a lymphatic fistula in a woman with chylous ascites after pelvic lymphadenectomy for cervical cancer. By injecting 3 ml of bilateral subcutaneous ICG between the first and second toes, they were able to locate the disrupted lymphatic duct on the right external iliac vessels laparoscopically using a NIR optic device, leading to complete resolution of symptoms. A similar approach was adopted by Fernandes *et al*.^28^ with a proximal thigh ICG injection followed by laparoscopic repair of the fistula.

The applications of ICG lymphography in gynecologic oncology are expanding, encompassing the assessment of lymphatic drainage and the visualization of lymphatic leakage. Even though the current quality of evidence is poor and limited to case reports, in the future, authors may use ICG lymphography to identify intraoperative lymphatic leakages and attempt to reduce the rates of chylous ascites and lymphoceles postoperatively. The main results of ICG lymphography are summarized in Table 1.

**Table 1:** This table provides a summary of ICG uses in the most common gynecologic oncology surgical applications: bowel surgery, ureteral surgery, and assessment of lymphatic system.

Field	Study	ICG application	Country	Study design	Sample size
LYMPHOGRAPHY	Unno *et al*., 2007^9^	Identify the presence of lymphatic disorders	Japan	Prospective	12
	Yamamoto *et al*., 2011^11^	Evaluate extremity lymphedema and indocyanine green lymphography patterns	Japan	Prospective	45
	Mihara *et al*., 2012^20^	Identify subcutaneous lymphatic vessels and vein	Japan	Prospective	6
	Akita *et al*., 2013^13^	Detect early-stage lymphedema, identify indocyanine green lymphography patterns and plan the surgical treatment	Japan	Prospective	50
	Chang *et al*., 2013^14^	Identity functional lymphatic vessels assessing lymphedema severity and selection of patients with extremity lymphedema for lymphovenous bypass	USA	Prospective	100
	Papadia *et al*., 2015^27^	Identify lymphatic fistula	Switzerland	Case report	1
	Ito *et al*., 2016^21^	Confirm patency of newly performed lympho-vascular anastomosis	Taiwan	Prospective	5
	Yamamoto *et al*., 2016^10^	Diagnose subclinic lower extremity lymphedema	Japan	Prospective	14
	Gentileschi *et al*., 2017^23^	Preoperative assessment of abdominal lymphatic drainage, to perform flap for after groin lymphadenectomy	Italy	Prospective	5
	Nguyen *et al*., 2017^15^	Staging lymphedema	USA	Prospective	42
	Fernandes *et al*., 2020^28^	Identify lymphatic fistula	Brazil	Case report	1
	Suami *et al*., 2022^16^	Diagnose lower limb lymphoedema and identity drainage regions	Australia	Prospective	326
	Caretto *et al*., 2022^18^	Identify lymphatic drainage and plan incision site to perform lymphatico-venular anastomosis	Italy	Retrospective	33
BOWEL	Moukarzel *et al*., 2020^29^	Identify postoperative anastomotic leakage	USA	Retrospective	133
	Nguyen *et al*., 2020^30^	Identify postoperative anastomotic leakage	Canada	Retrospective	100
	Jafari *et al*., 2021^31^	Identify postoperative anastomotic leakage	USA	Prospective	178
	Bizzarri *et al*., 2021^32^	Assess the ureteral anastomosis perfusion at time of pelvic exenteration for gynecologic malignancy	Italy	Prospective	15
URETER	Lee *et al*., 2013^33^	Identification of ureteral course and distinction between the healthy ureter from diseased tissue	USA	Prospective	7
	Cabanes *et al*., 2019^34^	Identification of ureteral course	Spain	Case series	16
	Mandovra *et al*., 2019^35^	Identification of ureteral course in laparoscopic surgery	India	Prospective	30
	Park & Farnam, 2015^36^	Identification of ureteral course in robotic surgery	USA	Case series	10

## Angiography

When administered intravenously, the ICG molecule quickly binds to plasma proteins, and intraoperative fluorescence is generally visible in less than a minute. It rapidly reaches the small arterial vessels that perfuse the observed organs, diffusing throughout the entire organ within a few minutes. The presence and quantification of fluorescence have provided surgeons with a valuable tool to assess tissue vascularization after a demolitive procedure. It also enables the recognition, based on the drug’s uptake patterns, of pathological tissues compared to healthy ones or specific structures to preserve or resect during surgery. In this section, the applications of angiography using ICG in gynecologic oncology will be analyzed.

### Bowel surgery

**Figure 1:**
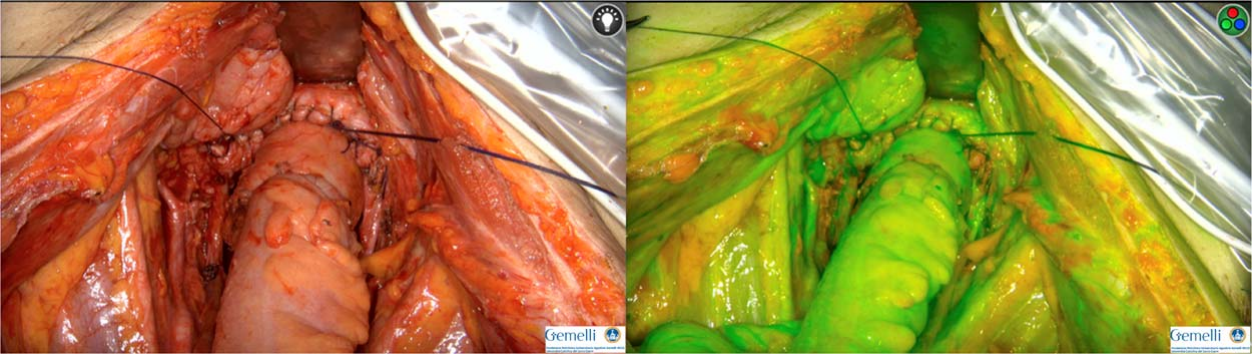
ICG Bowel Perfusion.

To achieve complete tumor clearance, bowel resections are often necessary in major oncological surgeries. Complications related to bowel anastomosis dehiscence are associated with high mortality rates due to sepsis. Unfortunately, the anastomosis with the highest risk of complications is the rectosigmoid one, which is the most common in gynecologic oncological surgery^37^. The most significant risk factors for anastomotic leakage are the presence of multiple bowel resections^38^, low body mass index and low preoperative albumin levels^39^. Furthermore, Costantini *et al*.^39^, in an extensive retrospective series of sigmoid-rectal resections for ovarian tumors, demonstrated that the surgical technique sparing the superior rectal artery is associated with a lower risk of postoperative leakage. Indeed, adequate vascularization is a crucial factor for the success of the anastomosis. The role of ICG fluorescence via intraoperative intravenous injection (Fig. 1) is well-established in reducing anastomotic-related complications in colorectal surgery^40^. Three RCTs were published by general surgeons. Two of them failed to show difference between patients who underwent perfusion assessment and those who underwent standard surgical techniques^31,41^. The third demonstrated that ICG-angiography significantly reduced the incidence of anastomotic leakage from 16.3 to 9.1% (*P*=0.04). In a subgroup analysis this difference was only significant for lower anastomoses^42^. A recent meta-analysis confirmed that ICG-angiography was protective against anastomotic leakage, with a pooled risk ratio of 0.67^43^. Nguyen *et al*. published a retrospective series of 100 gynecological cancer patients undergoing bowel resection. The vascular supply of the anastomoses was assessed with ICG. A proctoscope was used for low rectal resections, and an external camera was used for other resections. ICG assessment led to the revision of two anastomoses and one diverting ileostomy, with only one postoperative leakage^30^. In another large series of 410 gynecologic cancer patients, 133 underwent NIR angiography via proctoscopy to assess anastomotic perfusion during rectosigmoid resection and anastomosis. The postoperative anastomotic leakage rate was 1.5% in the NIR cohort compared to 4.7% in the non-NIR cohort. Although this difference was not statistically significant, the NIR group experienced significant advantages in terms of postoperative abscesses, interventional procedures, 30-day hospitalizations, and fewer protective ileostomies^29^. Main results of ICG bowel angiography are summarized in Table 1. Current evidence supporting the use of ICG in reducing complications related to colorectal anastomoses in gynecologic oncology is limited to large retrospective series. Although the results from general surgery are encouraging, they cannot be generalized since we operate in different settings, with different surgical techniques, patients with different prognosis, who undergo different adjuvant treatments. Therefore, randomized clinical trials in gynecological oncology are needed to evaluate the role of ICG in bowel surgery. On the one hand, the technique offers surgeons an intraoperative evaluation, allowing them to determine the actual need for a diverting ileostomy. On the other hand, objective cutoffs for assessing vascular supply are still lacking, and the evaluation remains strictly operator-dependent. The initial efforts to integrate artificial intelligence to make the evaluation of fluorescence as objective as possible, have come from general surgery. Cahill and colleagues developed machine learning algorithms to differentiate healthy tissues from benign polyps and cancerous lesions during colonoscopy. Briefly, they selected several regions of interest, focusing on the area related to endoluminal pathology. The ICG intensity curve was then analyzed by a machine learning model trained on a database of previously classified cases by a pathologist. This model was able to correctly diagnose 19 out of 20 cancers, achieving a sensitivity of 100% and a specificity of 92%. Additionally, a specifically developed video-tracking algorithm was applied to compensate for movements of the hand-held camera, patient respirations, and gas pressure variance^44^.The CLASSICA study protocol has been recently published. Authors aim to develop and validate a software able to characterize tissues based on ICG signal perfusion pattern analysis to differentiate benign and malignant colorectal polyps^45^. To date, currently published trial and meta-analysis in colorectal surgery suggest that ICG is more useful in preventing complications in high-risk patients. Therefore, further studies are required to demonstrate the true clinical role of NIR angiography, better defining the at-risk subpopulation who may benefit most from this technology. Finally, the integration of new surgical techniques involving vessel-sparing resection, combined with intraoperative angiography, could lead to significant improvements in the postoperative course of these patients.

### Pelvic exenteration

**Figure 2:**
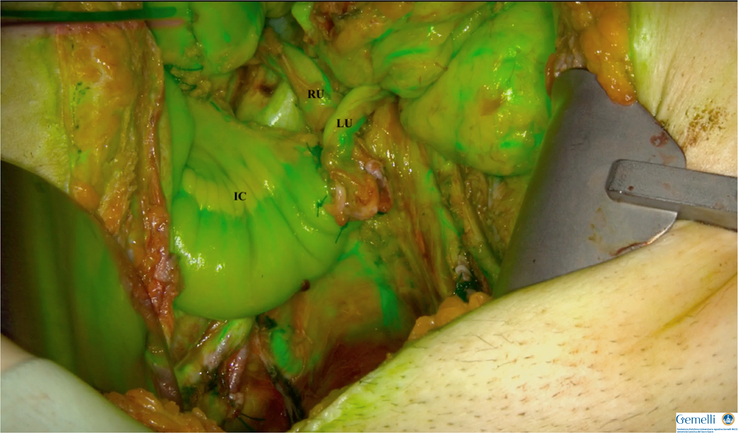
Uretero-ileal anastomosis vascularity assessed by ICG angiography.

Pelvic exenteration is the last curative option for patients with recurrent or persistent gynecological tumors who have previously undergone radiotherapy. This operation is associated with high morbidity^46^, with many complications related to urinary diversion^47^. Nowadays, several diversion techniques exist, but they are associated with a certain risk of ureteral fistulas and benign ureteral stenosis^48,49^. The ileal conduit is the most commonly used technique in gynecologic oncology, in which the ureters are sutured to an isolated segment of the ileum. Ureteral stenosis is predominantly related to an ischemic-inflammatory condition resulting from ureteral vascularization defects, which may be related to several factors (improper ureteral mobilization, previous radiotherapy, tension, or tightness of ureteral-enteric anastomoses). ICG can, therefore, be used to assess ureteral perfusion intraoperatively. A few studies have reported this method to assess the perfusion of ureteral-ileal anastomoses^50,51^, showing a reduced rate of benign ureteral stenosis in patients with better intraoperative ureteral perfusion. Bizzarri *et al*. proposed this approach in gynecologic oncology in a pilot study, in which intraoperative angiography was performed by intravenous injection to assess ureteral-ileal anastomoses, ileo-ileal anastomosis, and ileal conduit (Fig. 2). Due to the small number of patients, no significant correlations between the rate of stenosis and intraoperative findings were observed^52^. The evidence for utilizing ICG angiography in pelvic exenteration is limited but intriguing, especially given the high morbidity associated with these procedures. Therefore, further prospective studies are required to better understand whether ICG may improve morbidity outcomes in this setting.

### Ureteral perfusion and visualization

Gynecological surgeries are responsible for the largest proportion (52–82%) of iatrogenic ureteral injuries (UIs)^53^. Gynecologic oncology and advanced pelvic-reconstructive procedures are associated with UI more often than surgeries for other indications. UI results from laceration or transection, ligation or kinking with a suture, crushing with a clamp, thermal injury, or ischemia from devascularization^54^. A recent meta-analysis reported UI after 1.3–1.8% of radical hysterectomies^55^. On average, only 3–12% of UIs are detected intraoperatively when the patient could benefit from immediate repair^54,56^.

The use of ICG allows the identification of the ureteral course, potentially reducing the rate of iatrogenic UI or the proportion of unrecognized ureteral complications^56^.

The benefits of using ICG for intraoperative ureteral visualization and repair were first established a decade ago by Lee *et al*.^33^. Siddighi *et al*.^57^ applied this technique early on gynecologic surgeries that required intraoperative visualization of the ureter.

To visualize the ureteral course and prevent accidental damage, ICG can be administered under cystoscopy inside the ureters. This technique can be used either in laparoscopy or laparotomy^34^.

This technique has mainly been employed in minimally invasive settings. Mandovra *et al.* ^35^ and Park and Farnam^36^ demonstrated the feasibility of this technique by performing intraureteral injection of ICG in patients undergoing laparoscopic and robotic surgery. Ureteral fluorescence was achieved in all patients, without any intraoperative or postoperative ICG-related complications. The only differences between the patients concerned the intensity of the fluorescence, which differed for the amount of surrounding fat^35,36^.

Main results on ICG ureteral perfusion are reported in Table 1.

Again, the evidence provided by current literature is very limited to case reports and small series. Nevertheless, the use of NIR-ICG for intraoperative ureteral angiography and visualization appears to be a safe technique that can assist the surgeon in planning the best surgical strategy, avoiding intraoperative UIs, and appropriately selecting patients for ureteral stent placement. Furthermore, considering the high rate of complications associated with ureteral reimplantations in gynecologic oncology, especially in patients undergoing radiotherapy^58^, we suggest that ICG-angiography can be explored for assessing vascularization during ureteral reimplantation and for categorizing patients at higher risk of complications.

Further studies are necessary to validate this technique on a large-scale.

### Vaginal cuff perfusion

In gynecological cancers, radical surgery includes at least a total hysterectomy, after which a vaginal suture is needed. While in open approach the suture is performed abdominally, in minimally invasive settings the choice of a laparoscopic or vaginal closure relies on the operator’s choice, experience and attitude. Nevertheless, a possible complication is the vaginal cuff dehiscence (VCD). VCD occurs after 0.11–1.27% of laparoscopic, 0.45–1.64% of robotic-assisted, 0.05–0.13 of vaginal, and 0.02–0.38% of open hysterectomies^54^. Several risk factors have been identified (premenopausal status, vaginal atrophy, chronic steroid use, smoking habit, surgical-site infection, or supravaginal hematoma, vaginal brachytherapy, and chemotherapy), moreover extensive coagulation of cuff margins suture tension, suture spacing or suture layers play a role in vaginal cuff perfusion^54^. Intravenous ICG administration can be used to evaluate the vascularity of the vaginal cuff during surgery^59^.

In 2016, Beran *et al*. published two prospective studies conducted on forty patients undergoing total laparoscopic and robotic hysterectomy^60,61^. Authors concluded that the ICG assessment of the vaginal cuff vascularization is a valid possibility. If validated in prospective trials, a large-scale application of this technique might potentially allow surgeons to perform intraoperative revision of the suture to reduce postoperative rate of VCD. Moreover, a closer follow-up could be offered to high-risk patients.

### Uterine angiography

Uterine transposition is an innovative surgical technique proposed for the oncological management of young patients with rectal or anal cancer. The technique was first described by Ribeiro *et al*.^62^ in 2017. This procedure utilizes vascular anastomoses between the uterine arteries and the ovarian arteries. A possible complication of this technique is postoperative uterine necrosis. Kohler *et al*.^63^ recently described the use of repeated intravenous ICG injections to evaluate the uterine vascularization during uterine transposition to minimize the risk of postoperative necrosis.

While for nongynecological pelvic malignancies, uterus transposition is a reasonable option, for patients undergoing hysterectomy, future applications of uterus transplantation is a concrete perspective. Since the 2000s, several authors have described the procedure, with variable outcomes^64–66^. A crucial point for successful uterine transplantation is the patency of vascular anastomoses. In the past, the use of ICG for the assessment of post-uterine transplantation arterial flow has been described as part of the preclinical animal studies.^67,68^. The authors concluded that assessment of uterine perfusion and vessel stenosis, allows immediate intraoperative intervention to reduce the risk of postoperative failure.

Lastly, ICG uterine angiography has been employed to assess uterine vascularization in 20 radical trachelectomies, with or without uterine artery sparing. No differences have been observed in uterine fluorescence and pregnancy between groups^69^.

Given the rarity of these procedures, the quality of evidence supporting the use of ICG angiography is inevitably very limited and will likely remain scarce. Therefore, experimental use is strongly encouraged in the few surgeries performed to assess the potential utility of this form of angiography.

### Flap viability

The main treatment of vulvar cancer is the radical vulvectomy^70^. In case of large tumors or in recurrent, irradiated tumors, flap reconstruction is performed to fill the skin defects. Several methods exist to intraoperatively verify the vascularization of the flap, but they are expensive and not easily available. Instead, the use of ICG-angiography is easy and affordable, even in currently limited to few case reports in literature. Capozzi *et al*.^71^, described a series of 15 patients who underwent radical vulvectomy and surgical flaps for vulvar cancer. No surgical infection, dehiscence, or necrosis were recorded. Gentileschi *et al*. reported a case of complicated inguinal wound repair with a pedunculated left anterolateral thigh flap. An ICG angiography for flap viability was performed and no further complications were described. The patient was able to start radiotherapy 2 months after surgery^72^.

The ICG angiography could be considered for quickly assessing the viability of flap perfusion after surgery for gynecologic malignancies. An improvement in technology and technique is necessary to ensure the reliability of this method.

### Nerve visualization

Iatrogenic damage to pelvic nerves may occur during radical pelvic surgery, resulting in varying degrees of motor, sensory, and autonomic dysfunction. The nerves most frequently affected include the genitofemoral nerve, the obturator nerve, and the autonomic nerves, particularly the hypogastric nerve.

Over the years, several authors have described surgical techniques for performing radical hysterectomy while sparing nervous structures^73^.

Recently, the use of ICG has been proposed to prevent iatrogenic damage to nerve structures. Some authors have employed intravenous ICG administration to highlight the course of the hypogastric nerve and inferior hypogastric plexus, reporting no postoperative complications or autonomic dysfunction^74^.

Based on previous work using ICG for visualization of thoracic sympathetic nerves in lung cancer patients^75^, He *et al*.^76^ published the first prospective study to evaluate the feasibility and safety of intraoperative NIR fluorescence imaging to identify pelvic nerves during radical hysterectomy for cervical cancer. They enrolled 63 patients, achieving visualization of the obturator, genitofemoral, and hypogastric nerve courses in 100, 93.7, and 81% of cases, respectively.

ICG may prove useful and safe in real-time identification of pelvic nerves in patients undergoing radical hysterectomy for gynecological cancer, potentially reducing functional impairment related to nerve lesions. Nevertheless, given the limited data in the literature and the extensive number of surgeries performed by several referral centers, additional prospective randomized clinical trials are both feasible and needed. They will be necessary to offer a more precise evaluation of postoperative outcomes.

## ICG-guided resection

Minimally invasive approaches have various applications in selected scenarios in primary and recurrent settings of gynecological cancer^77–79^. Intraoperative fluorescence has the potential to overcome limitations of endoscopic surgery, such as absence of tactile feedback, providing a concrete support for successfully completing minimally invasive procedures. Moreover, in case of peritoneal involvement, particularly after systemic treatment identifying and differentiating tumors from scar tissue can challenge gynecologic oncologists, even in open surgery.

ICG has been used to intraoperatively identify anatomical structures or tumor locations, enabling precise surgical resection.

### Mesometrial and parametrial visualization

The ontogenetic theory of tumor development explains how carcinogenesis is linked to the reactivation of embryogenetic development. Therefore, tumors in the early stages develop within tissues belonging to the same embryogenetic compartment^80,81^. This theory led to surgical techniques that revolutionized the approach to certain neoplasms, such as rectal cancer^82^. Hockel adopted this compartmental resection approach for gynecological tumors^83^. Some authors described techniques to demarcate mesometrial or parametrial tissue with ICG fluorescence^84,85^. Kimmig *et al*. used ICG to highlight embryologically defined compartments in endometrial cancer surgery. Following the application of ICG on the middle and fundic myometrium, the Mullerian sub-compartment of the uterine body can be visualized. They performed a radical hysterectomy with mesometrial resection and lymphadenectomy in preoperatively identified intermediate/high-risk situations (56% of patients), while a simple extra fascial hysterectomy with BSO was performed in low-risk patients (44% of patients). The authors described a low loco-regional recurrence rate of 2.9% despite a meager rate of adjuvant radiotherapy^84^.

### Peritoneal metastasis visualization

ICG fluorescence may play a role in peritoneal metastases identification. The dye is administered intravenously and accumulates in the tumor cells, allowing real-time visualization during surgical procedures. This could potentially enable better identification and removal of peritoneal metastases, aiding the surgeon in achieving complete cytoreduction, particularly in surgery after neoadjuvant treatments (NACT). Furthermore, detecting a peritoneal involvement in apparent early-stage disease, could improve oncological outcomes by tailoring the therapeutic management. ICG fluorescence for detecting peritoneal metastases has been employed for patients with colorectal, hepatocellular, ovarian, and endometrial carcinoma^86^. Nevertheless, its applications in gynecologic oncology showed high rate of false positivity.

Although the preoperative imaging remains the gold standard in identifying tumor locations, there are several considerations justifying the implementation of fluorescent-guided surgery: 1) Peritoneal carcinomatosis is often inaccurately diagnosed by preoperative examinations, especially after neoadjuvant chemotherapy (NACT), and distinguishing fibrotic tissues from residual disease can be particularly challenging; 2) In the context of surgery for recurrence, intra-abdominal anatomy may be altered by adhesions and prior radiotherapy, making it difficult to identify lesions, even if easily visible on preoperative imaging; 3) Intraoperative findings often do not correlate with imaging findings, especially concerning very small lesions.

Tummers *et al*. analyzed ten patients undergoing surgery for suspected ovarian cancer. Eight metastatic lesions were detected, after ICG intravenous injection, in two patients. However, 13 nonmalignant lesions were also NIR-fluorescent, with a false-positive rate of 62%^87^.

Veys *et al*. identified a total of 108 peritoneal lesions in 20 patients with ovarian carcinoma, 15 of whom received NACT. The authors demonstrated that ICG is accurate in demonstrating peritoneal metastases in ovarian carcinoma but this ability is negatively affected by NACT. Furthermore, ICG fluorescence was unable to discriminate between benign and malignant lesions after NACT^88^.

Kreklau *et al*. described a series of three patients with single-site recurrence of gynecological tumors. In this series, the lesions were marked preoperatively with a CT-guided ICG injection 30 min before surgery. All lesions were successfully identified and resected^89^.

The use of ICG in the visualization of peritoneal metastases in gynecological cancers could potentially provide a significant advance in the surgical management of these malignancies; however, the low specificity shown in these series inevitably leads to seek an improvement of this technology as well as the use of more specific tracers for peritoneal metastases detection.

Although less known, other tracers have been employed for the recognition of peritoneal metastases in clinical trials. Folate receptor alpha (FRα) is highly expressed (80–90%) in Ovarian Cancer. OTL-38 is an FRα-targeted ligand bound to a cyanine dye^90^. 5-Aminolevulinic acid (ALA) is a prodrug that accumulates protoporphyrin-IX in the mitochondria of cancer cells; protoporphyrin-IX acts as a photosensitizer^91^. Recently, El-Swaify *et al*. published a systematic literature review on the clinical application of intraoperative fluorescent tracers for identifying peritoneal lesions during cytoreduction in ovarian cancer. The sensitivity, specificity, and positive predictive value for micrometastases detection of OTL-38 and 5-ALA at time of cytoreduction were 92.2 vs 79.8%, 67.3 vs 94.8%, and 55.8 vs 95.8%, respectively^92^. The most recent phase-3 trial (NCT03180307) demonstrated that 47 out of 109 OC patients underwent additional resections when OTL-38 fluorescence was activated after routine cytoreduction^93^. Additionally, ONM-100, a polymer conjugated to ICG, is a pH-sensitive, tumor acidosis-activated targeted fluorophore^90^. A phase-2 study (NCT04950166) recently completed enrollment, to assess the potential of ONM-100 in detecting peritoneal metastases (including ovarian cancer). ONM-100 offers advantages such as not being biomarker-related, avoiding issues related to heterogeneous expression, and exhibiting exceptional sensitivity in identifying peritoneal metastases in solid tumors^94^.

### Identification of peritoneal contamination in cervical cancer surgery

Minimally invasive surgery for cervical cancer has recently come under the spotlight after the publication of a randomized phase III trial showing impaired survival for cervical cancer operated with minimal invasive approach^95^. The most probable hypothesis is related to peritoneal contamination by cervical neoplastic cells, favored by gas flow during laparoscopic colpotomy^32^. Klapdor *et al*. proposed a proof of principle study for laparoscopic hysterectomy. ICG was applied on the cervical surface in 12 cases of minimally invasive hysterectomy for benign pathology. During colpotomy, images with white and NIR light were acquired to recognize the peritoneal spreading of cervical surface cells. Contamination was recognized in nine cases^96^. This approach might be considered an effective quality assessment tool for surgical techniques, even if the current quality of evidence is based on a single hypothesis-generating study.

### Detection of margin of resection

Preoperative examinations show the degree of neoplastic invasion, but this kind of information does not translate into real-time visualization of tumor margins intraoperatively. Currently, there is no validated intraoperative test able to provide accurate information on disease extension. Frozen sections are often altered by coagulation artifacts and display only a single spot of the resection margin. ICG administration may provide an instantaneous picture of infiltration and margins during surgery. In cervical, vaginal, and vulvar cancers, resection margin status is particularly critical for adjuvant radiotherapy choice, seriously affecting the economic burden and risk of complications^97^. Li *et al*.^98^ analyzed the correlation between the clinicopathological features of radical hysterectomy specimens from 48 IB1-IIA2 cervical cancer patients and NIR fluorescence imaging results. The NIR fluorescence imaging method was used to identify the extent of tumor invasion when radical hysterectomy specimens were taken. The specificity, sensitivity, and accuracy of NIR imaging were high (stromal deep infiltration predicted in 93.5% patients, margin infiltration detected in 100% patients). Nguyen-Xuan *et al*.^99^, reported two cases of vulvar-vaginal neoplasms, in which ICG-fluorescence was employed to determine limits of resection.

## Therapeutic applications

The therapeutic use of ICG as a theragnostic agent in gynecological cancers is emerging in numerous experimental publications. ‘Theranostic’ describes the combination of diagnostic properties and therapeutic effects of molecules targeting specific sites. Photothermal therapy (PDT) stands as a non-invasive tumor treatment method^100–103^, employing a photothermal agent that destroys tumor cells by converting light energy into heat. However, the limited depth of light penetration restricts the range of application for this technique^104^. Conversely, sonodynamic therapy (SDT) relies on ultrasound energy capable of targeting much deeper regions to activate local cytotoxicity^105,106^. Although the differences between PDT and SDT have not been fully elucidated, both methods seem to induce cytotoxic effects by generating Reactive Oxygen Species (ROS), leading to apoptosis and cell necrosis^107–110^. The combination of PDT/SDT and chemotherapy offers advantages from both treatment modalities^111^. Fluorescent nanoprobes are often employed due to their ability to achieve targeted imaging and synergistic therapy simultaneously, with ICG being one of the most frequently used molecules.

Nevertheless, ICG presents certain disadvantages, such as limited photostability, a high binding affinity to plasma proteins, and a tendency to aggregate in aqueous solutions, thereby restricting its applications^112^. Efforts have been made to overcome these issues, with several nanocarriers being employed to encapsulate ICG and form nanoparticles (NPs) to enhance its stability^113,114^.

In gynecologic oncology, the efficacy of these NPs in preclinical models of ovarian and cervical carcinoma has been analyzed. Nanocarriers used to encapsulate ICG often consist of polymers or combinations of polymers and lipids^115,116^. Other molecules with theragnostic properties such as Polifluorocarbonate^117–120^, chemotherapeutics agent like oxaliplatin^118,120,121^, taxol^119^, and doxorubicin^113^ can be encapsulated within polymeric or hybrid nanocarriers.

Chen *et al*. created a polymer-lipid hybrid NP aimed at improving the molecule’s stability, encapsulation efficiency, and targeting ability. These hybrid NPs were loaded with ICG and a polifluorocarbonate, with the lipid surface coated with the folate receptor. The folate receptor, potentially overexpressed on the surface of ovarian cancer cells while being expressed at low or negligible levels in normal tissues, holds promise as a biological target^117^.

Polifluorocarbonates serve as excellent oxygen carriers that increase oxygen concentration at the target site. Additionally, polifluorocarbonate-loaded NPs can produce microbubbles under laser irradiation, providing excellent contrast for ultrasound and photoacoustic imaging^122^.

Furthermore, ICG can induce ROS generation through PDT-SDT to achieve a cytotoxic effect^123^.

Other researchers have reported the construction of nanoparticles encapsulating a different molecule: the new ICG (IR-820)^124–126^. IR-820 not only exhibits similar optical and photothermal properties to ICG but also offers better stability and tissue retention^127^ ^128,129^.

In conclusion, these NPs capable of transporting ICG, chemotherapeutics, and other substances can be employed as dual-mode imaging guides with synergistic therapeutic effects. While these technologies in the field of gynecological oncology remain in the preclinical phase, promising results have been described regarding their potential for a tailored therapeutic approach for oncological patients.

## Safety and ethical considerations

ICG is an amphiphilic tri-carbocyanine dye, provided as a lyophilizate, requiring dissolution in water before injection. Once in the bloodstream, the dye swiftly binds to proteins, once metabolized by the liver, ICG is eliminated through the biliary system, exhibiting a half-life of 2–4 min. Used in medicine since the late fifties, ICG has shown very rarely reported side effects. Out of a total of 240 000 ICG administrations, only four intolerance reactions (1/60 000 cases) were reported^130^. Recently, Capasso *et al*. published an article on the safety of ICG in sentinel lymph node detection in endometrial research. Among 923 patients, 10 (1.1%) developed transient skin reactions in the subsequent 7 days, not conclusively linked to ICG use. None of these patients had a history of adverse reactions to iodinated contrast media^131^.

Adverse reactions to ICG are exceedingly rare, usually mild to moderate, but potentially life-threatening. Therefore, gynecological oncologists must be aware of possible complications when using this drug. When used off-label, it should be within approved clinical studies overseen by the referral ethics committee. Furthermore, when used as a diagnostic tool, the patient should be adequately informed about the possibility of false positives and negatives associated with the procedure, as well as the clinical and prognostic implications that may arise from such inaccuracies.

## Discussion

The realm of ICG fluorescence presents an intriguing landscape, offering a rich tapestry of anatomical insights that hold the potential to transform surgical approaches and postoperative care. While many of the applications discussed in this report are still at the experimental stage, and some remain in the preclinical phase, their ease of implementation, cost-effectiveness, and low toxicity profile make them compelling candidates for clinical integration. The incorporation of artificial intelligence into surgical practices addresses a central challenge in fluorescence assessment – the subjectivity inherent in interpretation. While quantifying fluorescence may not fundamentally alter surgical planning with respect to anatomical details, it plays a pivotal role in guiding functional assessments. Variations in fluorescence intensity, influenced by technical factors or camera proximity to the tissue, can introduce uncertainty into surgical decision-making. The evolution towards precise and detailed fluorescence quantification has the potential to elevate clinical outcomes and broaden the applicability of these techniques. In gynecological cancer surgeries, complications involving the bowel and ureter are relatively common, emphasizing the emerging need for a dependable perfusion assessment. Such an assessment could guide the decision to employ protective measures like diverting ileostomies or ureteral stent placements. To date, solid data favoring the intraoperative vascularity assessment by ICG come only from colorectal surgery trials^43^. Future gynecological studies should strive to incorporate rigorous objective perfusion assessment technologies. Another intriguing prospect lies in the integration of ICG into nanoparticles with multifunctional properties, capable of selectively delivering therapeutic benefits to tumor tissues while concurrently providing diagnostic insights^7^.

The significant limitation of this narrative review is that, except for bowel angiography and lymphography, which are already employed in clinical practice, most of the described applications are based on small case series or case reports. Consequently, the quality of the reported data is low and is insufficient to confidently determine the utility of these techniques. However, the ease of use and affordability of the method may facilitate the emergence of numerous studies based on ICG fluorescence.

The path forward invites further investigation into the feasibility and safety of these innovative therapeutic approaches. As technology continues to advance, there is a growing interest among surgeons in augmented reality. If these evolving technologies can harmoniously align with surgeons’ needs while remaining cost-effective, image-guided surgery may evolve from novelty to an integral component of precision and personalized surgical interventions. It promises to shed light on previously concealed aspects of tissues, anatomical structures, and physiological processes. The realization of the full potential of ICG-driven surgery will depend on clinical trials that rigorously assess its benefits, offering the potential to reshape surgical practices in a patient-focused manner.

## Ethical approval

Not applicable.

## Consent

Not applicable.

## Sources of funding

Not applicable.

## Author contribution

M.L., N.B., G.S., and F.F.: conceived the idea for the manuscript and led the writing and editing of the manuscript; F.M.C., R.W., D.Q., A.G., A.N., F.S., N.F., and A.F.: all reviewed, edited, and contributed to the final manuscript.

## Conflicts of interest disclosure

The author declares no conflicts of interest.

## Research registration unique identifying number (UIN)

Not applicable.

## Guarantor

Matteo Loverro and Nicolò Bizzarri.

## Data availability statement

Not applicable.

## Provenance and peer review

The paper was non-commissioned.
